# How do Chinese people perceive their healthcare system? Trends and determinants of public satisfaction and perceived fairness, 2006–2019

**DOI:** 10.1186/s12913-021-07413-0

**Published:** 2022-01-04

**Authors:** Yishan Zhu, Yuanyuan Li, Ming Wu, Hongqiao Fu

**Affiliations:** 1 National School of Development, Peking University, Beijing, China; 2 Department of Health Policy and Management, School of Public Health, Peking University Health Science Center, Beijing, China

**Keywords:** Public perception, Satisfaction, Fairness, China, Health system, Repeated cross-sectional survey

## Abstract

**Background:**

The public’s perception of the health system provides valuable insights on health system performance and future directions of improvement. While China’s health care reform was a response to people’s discontent in the health care system due to the lack of accessibility and affordability, little is known on changes in public perception of China’s health system. This paper examines trends in public perception of the health system between 2006 and 2019 and assesses determinants of public perception in China’s health system.

**Methods:**

Seven waves of the China Social Survey, a nationally representative survey, were used to examine trends in public satisfaction with health care and perceived fairness in health care. Chi-square tests were used to examine differences across waves. Logistic regression models were used to explore determinants of public perception, including variables on sociodemographic characteristics, health system characteristics, and patient experience.

**Results:**

Satisfaction with health care increased from 57.76% to 77.26% between 2006 and 2019. Perceived fairness in health care increased from 49.79% to 72.03% during the same period. Both indicators showed that the major improvement occurred before 2013. Sociodemographic characteristics are weakly associated with public perception. Financial protection and perceived medical safety are strongly associated with public perception, while accessibility is weakly associated with public perception. Patient experience such as perceived affordability and quality in the last medical visit are strongly associated with public perception of the health care system, while the accessibility of the last medical visit shows no impacts.

**Conclusion:**

Public satisfaction on health care and perceived fairness in health care in China improved over 2006–2019. The main improvement occurred in accordance with huge financial investments in public health insurance before 2013. Financial protection and perceived quality play significant roles in determining public perception, whereas accessibility and sociodemographic characteristics have limited influence on people’s perception of China’s health system. To achieve higher satisfaction and a higher sense of fairness in health care, China’s health system needs to continue its reforms on hospital incentives and integrated delivery system to control health expenditure and improve health care quality.

**Supplementary Information:**

The online version contains supplementary material available at 10.1186/s12913-021-07413-0.

## Background

Starting in the early 2000s, China initiated a series of healthcare reform measures to address the widespread public discontent with the inability of the health system to provide accessible and affordable healthcare services (commonly known as “kan-bing-nan, kan-bing-gui” in Chinese) [1–4]. First, the Chinese government launched three social health insurance schemes in early 2000s to cover urban and rural populations. The national coverage rate of social health insurance increased from less than 30% in 2003 to over 90% in 2009 [2, 3]. This increase was coupled with a large change in the health care financing structure. The share of out-of-pocket spending in total health expenditures decreased from 59.9% in 2000 to 37.5% in 2009 [5]. Between 2009 and 2011, the focus of healthcare reform was to expand health insurance coverage, to improve financial protection, and to increase fiscal investments in strengthening health care infrastructure [3]. By end of 2012, the national health insurance coverage rate exceeded 95% and the share of out-of-pocket spending dropped to 34.4% [1, 3]. From 2012 onward, China’s health system reform focused on restructuring the hospital-centric delivery system [3]. Policies on reforming public hospitals, establishing medical alliances, and payment reform were issued successively. Previous studies showed that China’s healthcare reform in the past 20 years achieved laudable progress in improving healthcare access and financial protection [3, 4, 6, 7]. Health care utilization increased in terms of outpatient visits and hospital admissions [3, 4]. From the perspective of financial protection, the incidence of catastrophic health expenditure and medical impoverishment have both decreased over the years [6, 7]. However, little is known on how the reforms have changed public perception of China’s healthcare system.

In the last 20 years, there is a growing interest in understanding public perception of the healthcare system since it has multiple uses. First, public perception is an important measure in evaluating health system performance, as adequate responsiveness is widely recognized as one of the core goals for the health system [8, 9]. Public perception metrics are increasingly used for international comparison of health system performance [10, 11]. Second, exploring determinants of public perception helps policymakers better understand the needs of the population and identify reform directions for improving the health system [12]. Lastly, eliciting public views brings legitimacy to policymaking by increasing transparency and accountability [13].

Existing research on public perception of the health system concentrates on studying public satisfaction and exploring its determinants. These studies find that sociodemographic characteristics [8, 14, 15], patient experience [16–19], and health system characteristics [16, 19, 20] are all associated with public satisfaction. Moreover, these studies show that the relationship between these factors and public satisfaction varies across countries. For example, the US population values “access to most-preferred care” much more than other high-income countries [20], while the quality of care has the largest impact on public satisfaction in South Korea [21]. In addition, several studies investigated other aspects of public perception of the health system, such as perceived fairness [22, 23], state involvement [24, 25], and public trust [26, 27].

There is a small body of China-specific studies on public perception of the health care system. Past research has examined satisfaction, perceived equality, and public trust in China’s health system [15, 28–30]. Some studies compare health system satisfaction with satisfaction in other social areas [31, 32]. However, these studies mainly focus on a short period. To the best of our knowledge, few studies have examined how public perception evolves with healthcare reform initiatives in a long period. Moreover, the determinants of public satisfaction and public trust studied in these studies are mostly sociodemographic characteristics, whereas the influences of health system characteristics are rarely discussed. We have a limited understanding of the relationship between health system characteristics and public perception in China.

The objective of this study is to describe temporal trends in public perception of China’s health system between 2006 and 2019 using satisfaction and perceived fairness as measures. Public satisfaction and perceived fairness are the main variables of interest in this study. Public satisfaction measures people’s attitude on the health system instead of people’s attitude on a specific health care visit. Perceived fairness is a subjective measure of people’s feelings on health care inequality. Together, they can provide an overall description of the public’s perception on the health system. In addition to describing the trends, we adopt a previous analytical framework of satisfaction with the health care system to examine determinants of public perception of the health system in China [21]. The framework identifies access, cost, and quality of care as the three factors that are associated with satisfaction of the health system. We use data from the China Social Survey to examine the relationship between the public perception and other health system performance metrics, including affordability, accessibility, and health care quality. The results of these statistical analyses reveal future directions for further improving China’s health system.

## Methods

### Data

The China Social Survey (CSS) was used to examine trends in public perception of the Chinese health system. It is a publicly available dataset, and the authors are not involved in the CSS design or fieldwork [33]. The CSS is a nationally representative repeated cross-sectional survey project initiated by the Chinese Academy of Social Sciences in 2006 and has been widely used in cross-sectional and trend analysis of China’s social and economic issues [34–37]. All participants were informed about the research questions and study objectives and written informed consents were obtained from all respondents [38]. A multi-stage, stratified, national probability sampling method was used to select and interview households, covering 151 counties from 30 provinces and autonomous regions in China. To ensure the quality of the survey, data collectors were trained via 3–5 days session and quality control measures were developed [39]. The survey was conducted every two or three years and as of date, seven waves of data are available, including the years of 2006, 2008, 2011, 2013, 2015, 2017, and 2019. The questionnaire in the CSS is consisted of a base module and several rotating modules. The base module is consistent across waves, including questions on demographic characteristics, socioeconomic status, and opinions on social issues (e.g., health care, education, housing, air pollution, corruption, etc.). Rotating modules vary across waves [39]. For example, in the wave of 2013, the questionnaire includes an additional module on patient experience for respondents who have visited a health care facility after 2000. However, in the other waves, no questions on patient experience were included. In addition, the question on perceived medical safety was part of the rotating module on social values which was covered in 2006, 2008, 2013 and 2017.

Table 1 shows summary statistics for the CSS data by waves. The sample size for each wave is 7061, 7139, 7036, 10,206, 10,243, 10,143, and 10,283, respectively. The average age of the sample is slightly over 40 years old with small standard deviation. Male consists of around half of the sample. The share of respondents who at least attended middle school increases gradually. Most of the respondents are married and employed across waves. Annual household income was relatively low and unstable in 2017, but the general trend was increasing from 19,581.24 RMB in 2006 to 93,942.54 RMB in 2019. People who are covered by public health insurance have more than doubled between 2006 and 2019. During the same period, the share of respondents who experienced unbearable health expenditures significantly decreased substantially. The average household income has experienced a great increase from 19,581 RMB to 93,942 RMB during this period.

**Table 1:** Summary statistics of the Chinese Social Survey, 2006–2019

	2006	2008	2011	2013	2015	2017	2019
	%	%	%	%	%	%	%
*Male*	49.74	49.90	50.50	50.77	50.83	50.86	50.85
*Urban hukou*	37.30	37.98	36.17	26.49	28.24	32.73	32.14
*Rural hukou (non-rural-to-urban migrant)*	50.11	50.84	44.70	46.07	41.13	36.14	38.89
*Rural hukou (rural-to-urban migrant)*	12.59	11.17	19.14	27.44	30.63	31.13	28.96
*Enrolled in middle school or above*	63.29	65.95	67.15	71.23	70.84	72.98	75.08
*Married or cohabiting*	79.94	77.59	76.31	77.94	79.41	77.41	76.64
*Employment status (Employed* = *1)*	72.90	72.78	70.33	72.88	70.12	64.89	67.23
*Covered by public health insurance (Yes* = *1)*	31.50	64.74	84.88	89.27	88.83	79.71	84.11
*The experience of unbearable health* *expenditure in the last year (Yes* = *1)*	45.07	37.59	28.16	29.20	33.23	32.37	30.50
*Self-rated social status (above average* = *1)*	45.14	47.94	52.08	48.77	41.55	34.67	47.06
*Perceived medical safety*	62.55	72.64	N/A	74.72	N/A	77.98	N/A
	mean	mean	mean	mean	mean	mean	mean
*Age*	40.88	41.25	42.60	39.30	41.27	41.89	41.89
	[0.19]	[0.20]	[0.24]	[0.16]	[0.15]	[0.15]	[0.15]
*Annual household income (RMB)*	19,581.24	29,163.98	56,376.15	63,657.69	70,199.59	68,127.94	93,942.54
	[459.32]	[659.46]	[2069.24]	[2255.11]	[1304.15]	[1455.16]	[2202.50]
Observations	*n* = 7061	*n* = 7139	*n* = 7036	*n* = 10,206	*n* = 10,243	*n* = 10,143	*n* = 10,283

Weighted percentages and means are included in the table; standard deviation for age and annual household income are reported in brackets. Annual household income is not adjusted by CPI

### Variables

The main variables of interest in this study are satisfaction with health care and perceived fairness of health care. Respondents were asked “Are you satisfied with the health care in your region?” A binary variable “satisfaction on health care” was constructed using this question, assigning “1” if the response is very satisfied or satisfied and “0” if the response is dissatisfied, very dissatisfied, or not sure. We classified “not sure” as “0” because the respondent does not exhibit a clear positive response. For perceived fairness in health care, respondents were asked “Do you think health care is fair in this society?” A binary variable “perceived fairness in health care” was constructed using this question. The variable equals “1” if the answer is relatively fair or very fair and “0” if the answer is relatively unfair, very unfair, or not sure.

Three sets of explanatory variables were included to explore the determinants of public perception. The first set is sociodemographic variables. Following the literature, the variables are age, gender, education level, marital status, employment status, migrant status, region, household income, and self-rated social status [14, 15, 29]. For education level, a dummy variable “middle school or above” was constructed. The variable equals “1” if a respondent at least attended middle school and “0” otherwise. Since migrant status and hukou status can largely influence the types of social health insurance schemes and health care utilization [40–42], these two indicators were used in the analysis. Based on individual hukou and migrant status, there are four types of residents: 1) residents with urban hukou living in urban area, 2) residents with urban hukou living in rural area, 3) residents with rural hukou living in urban area, 4) residents with rural hukou living in rural area. Among them, residents with urban hukou living in rural area were rare. Thus, respondents were divided into three groups: residents with urban hukou, residents who are migrant workers with rural hukou, and residents who live in rural area with rural hukou. These three groups differ in health insurance generosity and access to care so that they may have different perception on China’s health system. We use household income as a measure of a respondent’s economic status. This may not be a precise measure as opposed to disposable income of residents per capita. Therefore, we only use it as a control variable. We also divided the population by income quartiles so that we can examine whether heterogeneity of trends exists in subpopulations. Locations of respondents were grouped into three subcategories: East, West, and Central region. Self-rated social status was categorized into two groups: average or above and below average.

The second set of explanatory variables measures health system performance. Three aspects of health system performance, financial protection, access to health care resources, and quality of care, were used in this study [11, 21]. Insurance status, whether the family experienced unbearable health expenditure in the previous year, and the share of government health expenditure in total provincial health expenditure were used to reflect the level of financial protection. Experiencing unbearable health expenditure in the last year is a subjective proxy of financial protection. The question asks “Did you and your family encounter unbearably high medical expenditure in the last twelve months?” The number of hospital beds per 1,000 population and the number of medical professionals per 1,000 population in each province were used to reflect the accessibility of health care. Perceived medical safety was used as a subjective measure for the quality of care. Perceived medical safety was constructed as a binary variable that equals “1” if the respondent’s answer is relatively safe or very safe and equals “0” if it is relatively unsafe, very unsafe or do not know. Data on the share of government health expenditure in total health expenditure, the number of hospital beds per 1,000 population, and the number of medical professionals per 1,000 population were collected from the China Health Statistical Yearbooks [5]. In addition, social and economic factors, such as province level GDP per capita, percentage of province population over 65 years old, and province-level government spending per capita, were included as control variables.

The third set of variables are based on questions on patient experience in the 2013 wave. In this wave, respondents were asked about the experience of the recent visit to a healthcare facility since 2000. Questions include three aspects: accessibility, financial protection, and quality of care. Approximately 82% of respondents had visited a health care facility at least once after 2000 and then answered these questions. Respondents were first asked whether they experience problems of long travel distance, long waiting time, and high expenditure and how severe the problems are. For each problem, a binary variable was constructed and equals “1” if the problem is very severe or relatively severe and equals “0” if the problem is not severe, does not encounter the problem, or unsure. Respondents were asked to rate doctor attitude, doctor skills, doctor ethics, hospital environment, hospital equipment, and hospital order on a scale of 10, with “1” as very dissatisfied and “10” as very satisfied. Using these questions, a set of binary variables were constructed to measure the perceived quality. Each variable equals “1” if the respondent’s answer is 6 or higher and “0” otherwise.

### Statistical Analysis

A descriptive analysis was conducted to examine trends in public perception of China’s healthcare system between 2006 and 2019. Using cross-sectional weights for each wave, weighted variable means were presented to ensure that our results are nationally representative. Differences between survey years were calculated and chi-square tests were used to test for statistical significance. Moreover, the sample was divided into subpopulations by migrant status, region, self-rated social status, and household income quartiles to examine whether heterogeneous trends exist in subpopulations.

To further examine the independent effects of sociodemographic variables and health system performance on satisfaction and perceived fairness, logistic regressions were used. Two sets of explanatory variables were included in the primary analysis. The first set is sociodemographic variables, and the second set is variables of health system performance. Definitions of these variables are described in the previous subsection. We hypothesize that financial protection, accessibility, and quality of care are positively associated with public satisfaction and perceived fairness. Regressions were conducted separately for the waves of 2006, 2013, and 2017 to examine variations of determinants across years. Other waves were excluded because certain key variables such as perceived medical safety were missing. Additional Table 1 lists the variables used in analysis and variables missing in each wave. Province-level GDP per capita, share of population over 65 years old, and government spending per capita were included in regressions to control for social and economic factors. Odds ratios (OR) and the average marginal effect (AME) were reported for each covariate. Furthermore, using the additional information on patient experience in the 2013 wave, the relation between hospital experience and public perception of the health care system was explored. Sociodemographic variables and measures of health system performance were also included in the analysis. Additionally, dummies for facility level, facility type, and the year of the last medical visit were included in regressions as controls.

## Results

### Overall trends

Table 2 presents the trends of public perception of China’s health system between 2006 and 2019. The proportion of people who were satisfied with health care increased from 57.76% to 77.26% between 2006 and 2019. The increase was 11.79 percentage points between 2006 and 2013, whereas the change between 2013 and 2019 was 7.71 percentage points. It suggests that the major improvements in public satisfaction occurred when government investments were increasing and people experience reductions in out-of-pocket expenditures. The trend in perceived fairness of health care was similar to that in public satisfaction. The proportion of respondents who believed the health care was fair increased from 49.79% to 72.03% between 2006 and 2019 and most of the increase occurred between 2006 and 2013. Between 2006 and 2013, the improvement in perceived fairness in health care was 19.27 percentage points while the increase was merely 2.96 percentage points between 2013 and 2019.

**Table 2:** Public perception of the health system in China, 2006–2019

Year	Satisfaction with health care	Perceived Fairness of health care	Observations
	Mean (%) [95% CI]	Mean (%) [95% CI]	N
2006	57.76 [56.47–59.03]	49.79 [48.50–51.09]	7061
2008	71.12 [69.94–72.27]	66.75 [65.53–67.95]	7139
2011	67.39 [66.10–68.66]	N/A	7036
2013	69.55 [68.45–70.63]	69.06 [68.00–70.11]	10,206
2015	69.08 [68.09–70.06]	68.29 [67.29–69.27]	10,243
2017	71.92 [70.94–72.88]	72.78 [71.80–73.73]	10,143
2019	77.26 [76.34–78.15]	72.03 [71.07–72.96]	10,283
Difference			
2006–2013	11.79^a^	19.27^a^	
2013–2019	7.71^a^	2.96^a^	
2006–2019	19.50^a^	22.23^a^	

Weighted percentages and 95% confidence intervals are reported in the table. The 2011 wave of CSS did not include the question on perceived fairness of health care. ^a^, ^b^ and ^c^ denote statistical significance at the 1%, 5%, and 10% level, respectively

**Fig. 1:**
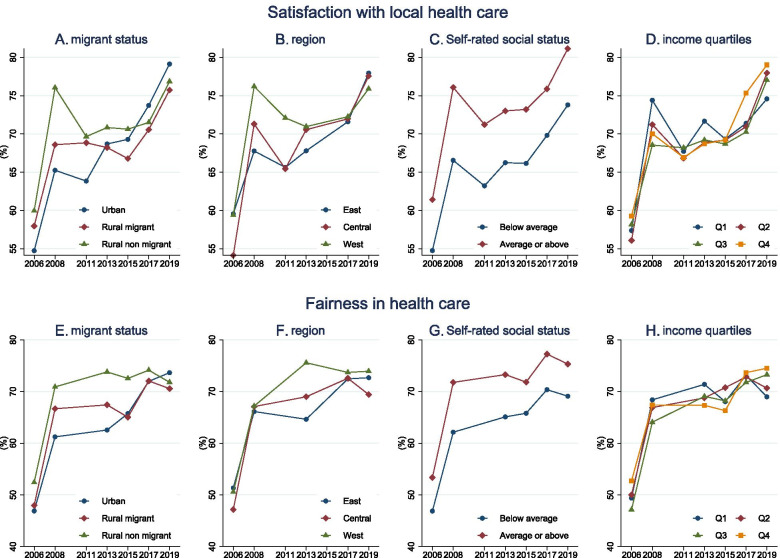
Trends in public perception by migrant status, region, social rank, and income quartiles, 2006–2019**.** Subfigure A, B, C, D show trends in satisfaction with local health care by migrant status, region, social rank, and income quartiles between 2006 and 2019. Subfigure E, F, G, H show trends in perceived fairness in health care by migrant status, region, social rank, and income quartiles between 2006 and 2019. Data source: The China Social Survey

Figure 1 shows trends in public satisfaction and perceived fairness by different subpopulation groups. While all subpopulations experienced improvements in public satisfaction and perceived fairness of the health system, the change was not consistent. Urban residents transformed from being the least satisfied population in 2006 to the most satisfied population in 2019, whereas rural migrants became the least satisfied population after 2015. People in the central region were significantly less satisfied with the health system than people in the east and the west regions in 2006. However, in 2019, the gaps between regions became smaller, with the west region having the lowest satisfaction rate. For people with different self-rated social statuses, those who view themselves as having an average or above social status were consistently more satisfied with health care across years. People in different household income quartiles do not exhibit consistent ranking in satisfaction.

As for fairness in health care, the proportion of rural non-migrants who felt the health system was fair was higher than that of rural migrants and urban residents in 2006. In 2019, rural migrants felt the health system was least fair compared to rural non-migrants and urban residents. Perceived fairness in health care was consistently higher in the west region compared to the east and the central region. People with higher self-rated social status consistently perceived the health system as fairer than people with lower self-rated social status. Perceived fairness in health care across household income quartiles did not exhibit a consistent pattern. In 2008 and 2013, wealthier groups perceived less fairness in health care than poorer groups, while in 2019 people with higher household income showed a higher sense of fairness.

### Determinants of public satisfaction and perceived fairness

Table 3 reports logistic regression results for determinants of public satisfaction in 2006, 2013, and 2017. The odds ratio, its associated 95% confidence interval, and the average marginal effect are reported for each regression. For factors influencing satisfaction with health care, age does not affect the odds of being satisfied across three waves. Enrollment in at least middle school decreases the odds of being satisfied in 2006. In 2013 and 2017, education does not affect people’s satisfaction. Male is less likely to be satisfied in 2006 and the effect was not statistically significant in 2013 and 2017. Married individuals are less likely to be satisfied in 2017. Being employed increase the odds of satisfaction in 2006 and 2013. Compared to people with urban hukou, rural non-migrants are more satisfied in 2006. People with higher self-rated social status are consistently more likely to be satisfied with health care. People in different household income quartiles do not exhibit differences in satisfaction across years. People in the west region are more likely to be satisfied than people in the east region in 2006. In 2013 and 2017, there are no statistically significant differences between the three regions.

**Table 3:** Determinants of public satisfaction in health care, 2006, 2013, 2017

	2006			2013			2017		
Variables	OR	95% CI	AME	OR	95% CI	AME	OR	95% CI	AME
**Sociodemographic**									
*Age*	0.992	0.960—1.025	-0.001^c^	1.014	0.984—1.044	0.002^a^	1.000	0.973—1.028	0.001^b^
*Middle school or above*	0.846^b^	0.737—0.972	-0.036^b^	0.921	0.804—1.055	-0.016	1.022	0.901—1.158	0.004
* Male*	0.896^c^	0.798—1.007	-0.024^c^	0.934	0.834—1.046	-0.013	0.981	0.879—1.094	-0.004
*Married*	0.994	0.834—1.183	-0.001	0.915	0.772—1.086	-0.017	0.841^b^	0.714—0.991	-0.032^b^
*Employed*	1.232^a^	1.063—1.429	0.046^a^	1.155^b^	1.008—1.322	0.029^b^	0.958	0.855—1.074	-0.008
*Rural non-migrant*	1.176^b^	1.011—1.369	0.035^b^	0.892	0.767—1.037	-0.023	0.947	0.826—1.085	-0.010
*Rural migrant*	1.101	0.918—1.321	0.021	1.006	0.877—1.155	0.001	0.911	0.799—1.039	-0.017
* Self-rated social status*	1.162^b^	1.029—1.312	0.032^b^	1.300^a^	1.161—1.457	0.052^a^	1.194^a^	1.066—1.337	0.033^a^
* Q2*	0.918	0.780—1.081	-0.018	0.926	0.788—1.089	-0.015	0.963	0.832—1.114	-0.007
* Q3*	0.964	0.815—1.141	-0.008	0.931	0.788—1.101	-0.014	0.912	0.788—1.057	-0.017
* Q4*	0.959	0.790—1.164	-0.009	0.910	0.759—1.090	-0.019	1.069	0.905—1.262	0.012
* Central*	1.051	0.820—1.346	0.011	0.804^b^	0.674—0.960	-0.042^b^	0.968	0.815—1.149	-0.006
* West*	1.566^a^	1.140—2.150	0.095^a^	0.672^a^	0.532—0.848	-0.078^a^	0.943	0.749—1.187	-0.011
**Financial protection**									
* Insured status*	1.283^a^	1.129—1.458	0.054^a^	1.574^a^	1.329—1.863	0.095^a^	1.249^a^	1.099—1.420	0.043^a^
* Experienced unbearable health expenditure*	0.679^a^	0.604—0.764	-0.085^a^	0.718^a^	0.638—0.807	-0.067^a^	0.697^a^	0.625—0.777	-0.070^a^
* Share of GHE in THE*	0.966^a^	0.946—0.987	-0.007^a^	1.002	0.988—1.016	0.000	0.997	0.982—1.013	-0.001
**Accessibility**									
* Hospital Beds/1000*	1.335^c^	0.972—1.834	0.062^c^	1.058	0.905—1.237	0.011	1.068	0.941—1.212	0.012
* Health professionals/1000*	1.020	0.848—1.225	0.004	1.081^a^	1.021—1.145	0.015^a^	0.978	0.883—1.083	-0.004
**Perceived quality**									
* Perceived medical safety*	3.399^a^	3.031—3.813	0.286^a^	2.494^a^	2.223—2.798	0.199^a^	2.886^a^	2.581—3.227	0.228^a^
**Province socioeconomic factors**									
* Log GDP per capita*	0.893	0.555—1.438	-0.024	0.382^a^	0.265—0.549	-0.190^a^	0.911	0.711—1.168	-0.018
* Population over 65 years old*	0.995	0.949—1.044	-0.001	1.091^a^	1.042—1.143	0.017^a^	0.993	0.952—1.035	-0.001
* Log Gov spending per capita*	0.707	0.387—1.292	-0.075	1.157	0.881—1.520	0.029	1.122	0.868—1.450	0.022
Observations	7,054			10,116			9,552		

^a^, ^b^ and ^c^ denote statistical significance at the 1%, 5%, and 10% level, respectively. *AME* is the average marginal effect. Data is weighted to yield nationally representative estimates. An increase of quartile corresponds to a progressive increase in household income quartiles. Q1, the urban area and the east region are used as reference groups

Financial protection significantly affects the probability of being satisfied. Insured individuals are more likely to be satisfied compared to uninsured individuals in 2006 (OR 1.283, 95% CI 1.129–1.458), 2013 (OR 1.574, 95% CI 1.329–1.863), and 2017 (OR 1.249, 95% CI 1.099–1.420). At the same time, having experienced unbearable health expenditure last year greatly decreases the odds of satisfaction with health care in 2006 (OR 0.679, 95% CI 0.604–0.764), 2013 (OR 0.718, 95% CI 0.638–0.807), 2017 (OR 0.697, 95% CI 0.625–0.777). However, the share of government health expenditure in total health expenditure at the provincial level does not affect satisfaction in 2013 and 2017. Number of hospital beds and medical professionals per capita at the provincial level is not associated with people’s satisfaction with health care. People who perceive medical care as safe are more likely to be satisfied with health care in 2006 (OR 3.399, 95% CI 3.031–3.813), 2013 (OR 2.494, 95% CI 2.223–2.798), and 2017(OR 2.886, 95% CI 2.581–3.227).

Table 4 reports logistic regression results for determinants of perceived fairness in health care in 2006, 2013, and 2017. Age slightly affects people’s perception of fairness in health care in 2013 and 2017. The effects of enrollment in at least middle school are inconclusive across three waves. Males are less likely to feel fair in health care than females in 2017 (OR 0.871, 95% CI 0.779–0.974). Being employed increase the odds of fairness in 2006 (OR 1.164, 95% CI 1.009–1.344). Compared to people with urban hukou, rural non-migrants are more likely to see health care as fair. The odds ratios are 1.272 (95% CI 1.100–1.4371) and 1.381 (95% CI 1.192–1.599) in 2006 and 2013, respectively. Rural migrants are indifferent from people with urban hukou in perceived fairness in 2006 and 2017. People with higher self-rated social status are consistently more likely to perceive health care in China as fair across years. People in different household income quartiles do not exhibit differences in perceived fairness in most years. People in the west region are more likely to have a sense of fairness in health care than people in the east region in 2013 (OR 1.286, 95% CI 1.027–1.611). However, the differences between regions are no longer statistically significant in 2017.

**Table 4:** Determinants of perceived fairness in health care, 2006, 2013, 2017

	2006			2013			2017		
Variables	OR	95% CI	AME	OR	95% CI	AME	OR	95% CI	AME
**Sociodemographic**									
*Age*	0.981	0.950—1.013	-0.001	0.956^a^	0.929—0.983	-0.001^c^	0.953^a^	0.927—0.981	-0.000
*Middle school or above*	0.999	0.872—1.144	-0.000	1.098	0.964—1.250	0.018	0.886^c^	0.780—1.006	-0.021^c^
*Male*	1.014	0.906—1.136	0.003	1.003	0.898—1.120	0.001	0.871^b^	0.779—0.974	-0.025^b^
*Married*	0.978	0.825—1.159	-0.005	1.155^c^	0.983—1.356	0.028^c^	0.876	0.743—1.033	-0.023
*Employed*	1.164^b^	1.009—1.344	0.035^b^	1.077	0.944—1.230	0.014	0.924	0.821—1.040	-0.014
*Rural non-migrant*	1.272^a^	1.100—1.471	0.055^a^	1.381^a^	1.192—1.599	0.064^a^	1.123	0.976—1.291	0.021
*Rural migrant*	1.041	0.871—1.243	0.009	1.237^a^	1.080—1.417	0.043^a^	1.056	0.924—1.208	0.010
* Self-rated social status*	1.114^c^	0.991—1.253	0.025^c^	1.359^a^	1.218—1.516	0.059^a^	1.284^a^	1.143—1.443	0.044^a^
* Q2*	0.999	0.853—1.171	-0.000	0.967	0.828—1.130	-0.006	0.961	0.827—1.117	-0.007
* Q3*	0.832^b^	0.705—0.982	-0.042^b^	0.966	0.823—1.134	-0.007	0.902	0.774—1.049	-0.018
* Q4*	1.030	0.855—1.241	0.007	0.937	0.788—1.116	-0.012	0.876	0.741—1.034	-0.024
* Central*	1.031	0.817—1.301	0.007	0.955	0.805—1.133	-0.009	0.920	0.770—1.098	-0.015
* West*	1.313^c^	0.973—1.773	0.062^c^	1.286^b^	1.027—1.611	0.048^b^	1.002	0.792—1.268	0.000
**Financial protection**									
* Insured status*	1.371^a^	1.212—1.551	0.072^a^	1.384^a^	1.174—1.633	0.065^a^	1.292^a^	1.133—1.472	0.047^a^
* Experienced unbearable health expenditure*	0.710^a^	0.633—0.796	-0.079^a^	0.757^a^	0.676—0.848	-0.055^a^	0.611^a^	0.547—0.682	-0.092^a^
* Share of GHE in THE*	0.960^a^	0.940—0.980	-0.009^a^	0.985^b^	0.972—0.998	-0.003^b^	0.994	0.979—1.009	-0.001
***Accessibility***									
* Hospital beds/1000*	0.985	0.736—1.319	-0.003	0.791^a^	0.680—0.921	-0.045^a^	1.022	0.898—1.164	0.004
* Health professionals/1000*	0.982	0.828—1.164	-0.004	1.105^a^	1.045—1.169	0.019^a^	0.965	0.872—1.069	-0.006
**Perceived quality**									
* Perceived medical safety*	2.905^a^	2.591—3.256	0.253^a^	3.195^a^	2.854—3.576	0.254^a^	3.580^a^	3.196—4.009	0.270^a^
**Province socioeconomic factors**									
* Log GDP per capita*	0.790	0.503—1.239	-0.054	0.351^a^	0.248—0.498	-0.202^a^	0.808	0.626—1.042	-0.038
* Population over 65 years old*	0.974	0.931—1.018	-0.006	1.062^b^	1.014—1.112	0.012^b^	1.015	0.972—1.060	0.003
* Log Gov spending per capita*	1.357	0.752—2.449	0.069	1.045	0.801—1.364	0.009	1.116	0.857—1.453	0.020
Observations	7,054			10,106			9,524		

^a^, ^b^ and ^c^ denote statistical significance at the 1%, 5%, and 10% level, respectively. *AME* is the average marginal effect. Data is weighted to yield nationally representative estimates. An increase of quartile corresponds to a progressive increase in household income quartiles. Q1, urban area and the east region are used as reference groups

Financial protection is strongly associated with perceived fairness in health care. Being insured increases the odds of a sense of fairness in 2006 (OR 1.371, 95% CI 1.212–1.551), 2013 (OR 1.384, 95% CI 1.174–1.633), and 2017 (OR 1.292, 95% CI 1.133–1.472), respectively. The experience of unbearable health expenditure in the last year decreases the likelihood of feeling fair in 2006 (OR 0.710, 95% CI 0.633–0.796), 2013 (OR 0.757, 95% CI 0.676–0.848), and 2017 (OR 0.611, 95% CI 0.547–0.682). Similar to the results on public satisfaction, having one more bed per 1000 population and having one more medical professional per 1000 population do not affect perceived fairness in most cases. But people who perceive medical care as safe are more likely to feel fair in health care in 2006 (OR 2.905, 95% CI 2.591–3.256), 2013 (OR 3.195, 95% CI 2.854–3.576), and 2017 (OR 3.580, 95% CI 3.196–4.009), respectively.

**Fig. 2:**
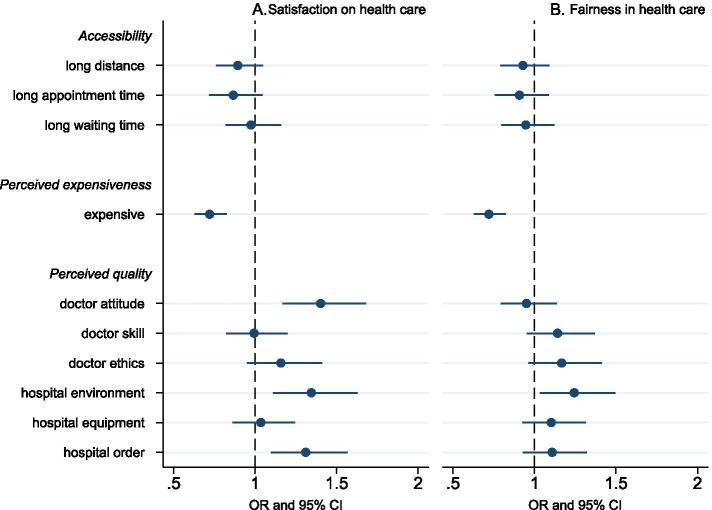
The association between patient experience and public perception, 2013**.** Logistic regression results on the relationship between patient experience and public perception in 2013. OR and 95% CI are shown in this figure. Panel A shows the association between patient experience and satisfaction with health care. Panel B shows the effect of patient experience on perceived fairness in health care. Sociodemographic variables and measures of health system performance in Tables 3 and 4 are included in the analysis. Additionally, we control for facility level, facility type, the year of the last medical visit, provincial GDP per capita, share of population over 65 years old, and government spending per capita. This figure only reports results on variables of patient experience and results on other variables are shown in Additional Table 2

Using cross-sectional data in 2013, the association between patient experience and public perception of the health system is examined. Figure 2 and Additional Table 2 show that among problems encountered during the last visit, accessibility measures, including long travel distance, difficulty in scheduling an appointment, and long waiting time do not influence people’s satisfaction and perceived fairness. However, the affordability measure (perceived expensiveness on the last medical visit) decreases the odds of satisfaction (OR 0.721, 95% CI 0.629–0.826) and perceived fairness (OR 0.720, 95% CI 0.629–0.824). In addition, measures on perceived quality in the last visit are also associated with people’s perception of the health system. Ratings on doctor’s attitude (OR 1.402, 95% CI 1.169–1.681), hospital environment (OR 1.345, 95% CI 1.111–1.628), and hospital order (OR 1.310, 95% CI 1.096–1.567) are positively associated with general satisfaction on health care. Satisfaction with the hospital environment is positively correlated with people’s sense of fairness in health care (OR 1.244, 95% CI 1.033–1.497).

## Discussion

In this study, we show that public satisfaction and perceived fairness in health care have improved over 2006–2019, with slower improvements in the second half of the period. While gaps still exist between subpopulations, all subgroups have experienced improvements. In terms of determinants of public perception on health care, we show that most variables on sociodemographic characteristics are weakly associated with satisfaction and perceived fairness. Moreover, we find that affordability and perceived quality play significant roles in determining public perception, while accessibility does not affect people’s perception of the health system much. The results on patient experience consolidate this finding. Regression estimates show that perceived affordability and quality in the last medical visit are strongly associated with patients’ perception of the health system. However, accessibility measures of the last medical visit are not important factors in determining public perception of health care.

The overall trend of improvement in public satisfaction and perceived fairness between 2006 and 2019 is concurrent with substantial progress in improving financial protection and accessibility during the same period [2, 3]. However, it should be noted that the major improvements in satisfaction with health care and perceived fairness were achieved between 2006 and 2012 rather than between 2013 and 2019. When we put the timeline into the context of China’s health system reform, we see that rapid improvements in public perception happened during the period when the government massively increased fiscal investments into the healthcare sector and expanded health insurance coverage. In contrast, public perception of the health system remained largely unchanged when the government started its structural reform in the healthcare delivery system [43]. This result is consistent with previous findings that improvements in financial protection and accessibility were mainly achieved in the earlier stage of healthcare reform [7]. All these findings imply that the effects of reforming the hospital-centric delivery system are neither widespread nor obvious. As shown in Additional Fig. 1, a rising share of respondents regards the health care issue as one of the top-three concerned social issues after 2013.

Our findings on the relationship between the socioeconomic variables and public perception are similar to previous cross-sectional studies of China [15, 29]. Most of the variables on sociodemographic characteristics except self-rated social status are weakly associated with public perception. However, the population with better sociodemographic status in China still have better access, financial protection, and healthcare quality [2, 3]. The small differences across subpopulations suggest that public perception of the health system is also influenced by their expectations [8, 15, 16].

Compared to sociodemographic characteristics, variables on health system performance are the main determinants of public perception of health care. We find that affordability of health care and perceived quality are much more important than the accessibility of health care in influencing people’s perception. The results on patient experience support this finding. It suggests that people in China are mostly concerned with financial barriers and quality. Despite universal coverage of health insurance and great improvements in financial protection, China is still facing a high incidence of catastrophic health expenditures and medical impoverishment, compared with other countries [44, 45]. Rapid growth in health expenditure caused by distorted provider incentives largely offsets the benefits of universal health insurance coverage. As for the quality of care, the limited progress in improving healthcare quality during China’s health system reform certainly cannot meet people’s rising expectations [46]. The importance of further reducing financial barriers and improving quality has been recognized by policymakers in China. Initiatives on building medical center hubs and controlling health expenditure growth by centralized procurement and payment reforms are recent examples of measures taken by the Chinese government to tackle these problems. Our result on accessibility shows discrepancies to the international literature. For example, a study on hospitals in Iran shows that travel time and waiting time scores the lowest among all dimensions of health system responsiveness, whereas our study shows travel time and waiting time are not significant determinants of satisfaction [47]. This is likely attributable to the fact that basic health care is relatively accessible in China. In 2018, 89.9% of Chinese households can access the nearest medical service within 15 min of transportation, and the average waiting time for a hospital admission is merely 1.5 days [48]. Another explanation on why accessibility is not important in China is that accessibility does not reflect whether people receive their most preferred care. For example, a study shows that access to most-preferred care, influenced by the wide variation in health insurance coverage and generosity, is important to satisfaction in the United States, whereas access itself is less important [20]. Similarly, the Chinese population may be already satisfied with the accessibility of basic health care, and they hope to receive better financial protection to access high quality health care.

Our study has several limitations. First, we are unable to distinguish satisfaction in access, financial protection, and quality of care, nor are we able to identify detailed aspects of system performance, such as appropriateness, continuity, and effectiveness. People may respond the questions with a certain aspect of health system performance in mind but our analysis could not reflect the details. This would require a series of questions, which may be a combination of quantitative and qualitative questions, to unveil people’s opinion and it would lead to a deeper understanding of health system performance.

Second, the relationship between patient experience and public satisfaction is drawn from the 2013 wave. We are unsure whether it can fully reflect the most recent views. This analysis is used to validate our findings that affordability of health care and perceived quality are much more important than the accessibility of health care in influencing people’s perception. Therefore, while the data on patient experience is not from a recent survey, it does not affect our main conclusion.

Third, we cannot use our results on public satisfaction without caution for international comparison. The CSS does not use the same questionnaire of the 2010 Commonwealth Fund International Health Policy Survey that is widely used in developed countries. In addition, the understanding of the wordings in the questions may be different across respondents. This means the trends rather than the numbers should be the main take away of this research. If we want to understand the level of satisfaction in China, we may need standardized questionnaires across countries to perform international comparison.

## Conclusions

This study shows that public satisfaction with health care and perceived fairness in China’s health system have improved between 2006 and 2019. The largest improvement occurred before 2013 when the government greatly increased its investments in health care financing. The trends of public satisfaction and perceived fairness in health care flattened after 2013. We also find that financial protection and perceived quality of care are important factors in determining the public’s perception of China’s health system. Our study contributes to a more comprehensive evaluation of China’s health system reform, as prior research focused on objective wellbeing measures and did not examine people’s satisfaction with the health system.

Our findings are relevant to the ongoing health system reform in China. We show that accessibility is no longer an important determinant of satisfaction and fairness in health care. To improve people’s satisfaction and sense of fairness in health care, policymakers should shift the focus to ensuring financial protection and providing health care services of high quality. From an operational viewpoint, it is important to deepen the reforms of health delivery system, especially public hospital reform and construction of an integrated delivery system, so that people can access health care services of higher quality at lower cost.

## Supplementary Information

**Additional file 1:** **Table S1.** Variables used in analysis.  **Table S2. **Patient experience and public perception, 2013. **Figure S1. **Top three social issues, 2006-2019.

## Data Availability

The data analyzed in the current study, the China Social Survey, is available at http://css.cssn.cn/css_sy/xmjs/.

## Abbreviations

CSS: China Social Survey
OR: Odds Ratio
AME: Average Marginal Effect
CI: Confidence Interval
