# Supplementary material for: How do Chinese people perceive their healthcare system? Trends and determinants of public satisfaction and perceived fairness, 2006–2019
Source: BMC Health Serv Res. 2022 Jan 4;22:22. doi: 10.1186/s12913-021-07413-0 (PMC8725557; doi:10.1186/s12913-021-07413-0)
Supplement: Supplementary file 1 — Additional file 1: Table S1. Variables used in analysis. Table S2. Patient experience and public perception, 2013. Figure S1. Top three social issues, 2006-2019. [file 12913_2021_7413_MOESM1_ESM.docx]

**Additional Files**

Additional Table 1 Variables used in analysis

Additional Table 2 Patient experience and public perception, 2013

Additional Figure 1 Top three social issues, 2006-2019.

**Additional Table 1 Variables used in analysis**

| **Variables** | **2006** | **2008** | **2011** | **2013** | **2015** | **2017** | **2019** |
| --- | --- | --- | --- | --- | --- | --- | --- |
| **Main dependent variables** |  |  |  |  |  |  |  |
| Satisfaction | Y | Y | Y | Y | Y | Y | Y |
| Perceived fairness | Y | Y | Missing | Y | Y | Y | Y |
| **Sociodemographic** |  |  |  |  |  |  |  |
| Age | Y |  |  | Y |  | Y |  |
| Gender | Y |  |  | Y |  | Y |  |
| Education | Y |  |  | Y |  | Y |  |
| Marriage | Y |  |  | Y |  | Y |  |
| Employment | Y |  |  | Y |  | Y |  |
| Urban/Rural | Y | Y | Y | Y | Y | Y | Y |
| Migrant/non-migrant | Y | Y | Y | Y | Y | Y | Y |
| Self-rated social status | Y | Y | Y | Y | Y | Y | Y |
| Annual household income | Y | Y | Y | Y | Y | Y | Y |
| Region | Y | Y | Y | Y | Missing | Y | Y |
| **Province socioeconomic factors** |  |  |  |  |  |  |  |
| Log GDP per capita | Y |  |  | Y |  | Y |  |
| Population over 65 years old | Y |  |  | Y |  | Y |  |
| Log Gov spending per capita | Y |  |  | Y |  | Y |  |
| **Financial proteciton** |  |  |  |  |  |  |  |
| Insurance status | Y |  |  | Y |  | Y |  |
| Experienced unbearable health expenditure | Y |  |  | Y |  | Y |  |
| Share of GHE in THE | Y |  |  | Y |  | Y |  |
| **Accessibility** |  |  |  |  |  |  |  |
| Hospital beds/1000 | Y |  |  | Y |  | Y |  |
| Health professionals/1000 | Y |  |  | Y |  | Y |  |
| **Perceived quality** |  |  |  |  |  |  |  |
| Perceived medical safety | Y |  | Missing | Y | Missing | Y | Missing |

**Note:** If the variable was used in either descriptive analysis or regression analysis in a specific wave, it is checked with a “Y”. If the variable was missing in the dataset or if it was not collected in a specific wave, we mark the cell as “Missing”. An empty cell indicates the variable is available but not used in anlaysis.

**Additional Table 2 Patient experience and public perception, 2013**

|  | (1) | (2) | (3) | (4) | (5) | (6) | (7) | (8) |
| --- | --- | --- | --- | --- | --- | --- | --- | --- |
|  | **Satisfaction with health care** | | | | **Perceived fairness in health care** | | | |
| VARIABLES | OR | OR 95% CI | AME | AME 95% CI | OR | OR 95% CI | AME | AME 95% CI |
|  |  |  |  |  |  |  |  |  |
| **Sociodemographic** |  |  |  |  |  |  |  |  |
| *age* | 1.010 | 0.976 - 1.045 | 0.001** | 0.000 - 0.003 | 0.954*** | 0.923 - 0.985 | -0.001** | -0.002 - -0.000 |
| *middle school or above* | 0.938 | 0.803 - 1.095 | -0.012 | -0.041 - 0.017 | 1.148* | 0.990 - 1.331 | 0.026* | -0.002 - 0.054 |
| *male* | 0.944 | 0.828 - 1.076 | -0.011 | -0.035 - 0.014 | 1.066 | 0.939 - 1.211 | 0.012 | -0.012 - 0.036 |
| *married* | 0.929 | 0.764 - 1.130 | -0.014 | -0.050 - 0.022 | 1.254** | 1.042 - 1.509 | 0.043** | 0.007 - 0.079 |
| *employed* | 1.077 | 0.923 - 1.257 | 0.014 | -0.015 - 0.043 | 1.045 | 0.901 - 1.214 | 0.008 | -0.020 - 0.036 |
| *rural non-migrant* | 0.837* | 0.698 - 1.004 | -0.033* | -0.067 - 0.001 | 1.375*** | 1.160 - 1.631 | 0.061*** | 0.028 - 0.094 |
| *rural migrant* | 0.961 | 0.818 - 1.130 | -0.007 | -0.037 - 0.022 | 1.267*** | 1.083 - 1.482 | 0.046*** | 0.015 - 0.076 |
| *Self-rated social status* | 1.207*** | 1.061 - 1.374 | 0.035*** | 0.011 - 0.060 | 1.278*** | 1.127 - 1.448 | 0.046*** | 0.023 - 0.069 |
| *Q2* | 0.900 | 0.750 - 1.079 | -0.020 | -0.053 - 0.014 | 0.953 | 0.798 - 1.139 | -0.009 | -0.042 - 0.024 |
| *Q3* | 0.960 | 0.796 - 1.157 | -0.008 | -0.042 - 0.027 | 0.914 | 0.761 - 1.097 | -0.017 | -0.051 - 0.017 |
| *Q4* | 0.882 | 0.719 - 1.082 | -0.023 | -0.062 - 0.015 | 0.906 | 0.742 - 1.107 | -0.018 | -0.056 - 0.019 |
| *Central* | 0.831* | 0.676 - 1.023 | -0.034* | -0.071 - 0.004 | 1.016 | 0.837 - 1.233 | 0.003 | -0.035 - 0.041 |
| *West* | 0.686*** | 0.521 - 0.904 | -0.070*** | -0.122 - -0.019 | 1.431*** | 1.104 - 1.854 | 0.066*** | 0.019 - 0.113 |
| **Financial protection** |  |  |  |  |  |  |  |  |
| *Insured status* | 1.642*** | 1.334 - 2.021 | 0.099*** | 0.055 - 0.142 | 1.358*** | 1.112 - 1.658 | 0.060*** | 0.019 - 0.100 |
| *Experienced unbearable health expenditure* | 0.822*** | 0.716 - 0.943 | -0.037*** | -0.064 - -0.011 | 0.827*** | 0.724 - 0.944 | -0.036*** | -0.062 - -0.011 |
| *Share of GHE in THE* | 1.009 | 0.993 - 1.025 | 0.002 | -0.001 - 0.005 | 0.986* | 0.972 - 1.001 | -0.003* | -0.005 - 0.000 |
| **Accessibility** |  |  |  |  |  |  |  |  |
| *Hospital beds/1000* | 1.051 | 0.875 - 1.264 | 0.009 | -0.025 - 0.044 | 0.775*** | 0.650 - 0.925 | -0.048*** | -0.081 - -0.015 |
| *Health professional/1000* | 1.085** | 1.018 - 1.157 | 0.015** | 0.003 - 0.027 | 1.099*** | 1.033 - 1.169 | 0.018*** | 0.006 - 0.029 |
| **Perceived quality** |  |  |  |  |  |  |  |  |
| *Perceived medical safety* | 2.145*** | 1.875 - 2.454 | 0.156*** | 0.127 - 0.184 | 2.779*** | 2.437 - 3.169 | 0.215*** | 0.186 - 0.244 |
| **Hospital characteristics** |  |  |  |  |  |  |  |  |
| *public* | 1.354*** | 1.107 - 1.655 | 0.057*** | 0.019 - 0.094 | 1.297** | 1.064 - 1.581 | 0.049*** | 0.012 - 0.086 |
| *secondary* | 1.037 | 0.887 - 1.212 | 0.007 | -0.022 - 0.036 | 1.077 | 0.926 - 1.253 | 0.014 | -0.014 - 0.042 |
| *tertiary* | 1.037 | 0.861 - 1.248 | 0.007 | -0.028 - 0.041 | 0.886 | 0.745 - 1.055 | -0.023 | -0.055 - 0.010 |
| **Accessibility (last visit)** |  |  |  |  |  |  |  |  |
| *long distance* | 0.894 | 0.761 - 1.050 | -0.021 | -0.051 - 0.009 | 0.929 | 0.792 - 1.090 | -0.014 | -0.044 - 0.016 |
| *long appointment time* | 0.866 | 0.717 - 1.046 | -0.027 | -0.062 - 0.008 | 0.907 | 0.757 - 1.088 | -0.018 | -0.052 - 0.016 |
| *long waiting time* | 0.974 | 0.818 - 1.160 | -0.005 | -0.038 - 0.028 | 0.946 | 0.798 - 1.122 | -0.010 | -0.042 - 0.022 |
| **Affordability (last visit)** |  |  |  |  |  |  |  |  |
| *Perceived expensive* | 0.721*** | 0.629 - 0.826 | -0.061*** | -0.087 - -0.036 | 0.720*** | 0.629 - 0.824 | -0.061*** | -0.087 - -0.036 |
| **Quality (last visit)** |  |  |  |  |  |  |  |  |
| *doctor attitude* | 1.402*** | 1.169 - 1.681 | 0.063*** | 0.029 - 0.097 | 0.950 | 0.795 - 1.137 | -0.010 | -0.043 - 0.024 |
| *doctor skill* | 0.993 | 0.823 - 1.199 | -0.001 | -0.036 - 0.034 | 1.142 | 0.952 - 1.369 | 0.025 | -0.009 - 0.059 |
| *doctor ethics* | 1.158 | 0.951 - 1.411 | 0.027 | -0.009 - 0.064 | 1.167 | 0.964 - 1.413 | 0.029 | -0.007 - 0.065 |
| *hospital environment* | 1.345*** | 1.111 - 1.628 | 0.055*** | 0.020 - 0.091 | 1.244** | 1.033 - 1.497 | 0.041** | 0.006 - 0.075 |
| *hospital order* | 1.310*** | 1.096 - 1.567 | 0.051*** | 0.017 - 0.084 | 1.108 | 0.929 - 1.322 | 0.019 | -0.014 - 0.052 |
| *hospital equipment* | 1.035 | 0.861 - 1.244 | 0.006 | -0.028 - 0.041 | 1.103 | 0.924 - 1.316 | 0.018 | -0.015 - 0.051 |
| **Province socioeconomic factors** |  |  |  |  |  |  |  |  |
| *Log GDP per capita* | 0.367*** | 0.242 - 0.556 | -0.188*** | -0.265 - -0.110 | 0.396*** | 0.267 - 0.589 | -0.173*** | -0.247 - -0.099 |
| *Population over 65 years old* | 1.094*** | 1.036 - 1.155 | 0.017*** | 0.007 - 0.027 | 1.061** | 1.005 - 1.119 | 0.011** | 0.001 - 0.021 |
| *Log Gov spending per capita* | 1.186 | 0.861 - 1.634 | 0.032 | -0.028 - 0.092 | 1.189 | 0.883 - 1.599 | 0.032 | -0.023 - 0.088 |
|  |  |  |  |  |  |  |  |  |
| Observations | 8,134 | | | | 8,124 | | | |

Note: *** p<0.01, ** p<0.05, * p<0.1 AME is the average marginal effect. The year of the last medical visit is also controlled. Data is weighted to yield nationally representative estimates. An increase of quartile (Q1, Q2, Q3 to Q4) corresponds to a progressive increase in household income quartiles. Q1, urban area and the east region are used as reference groups.


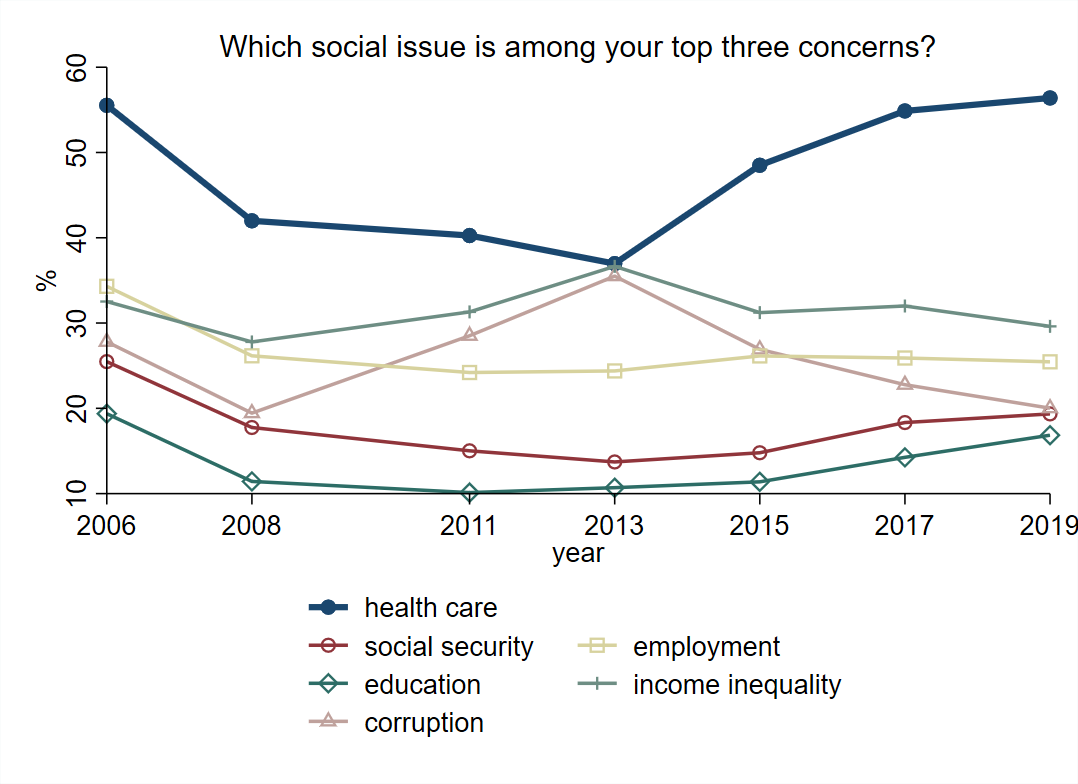


**Additional Figure 1 Top three social issues, 2006-2019.** Notes: Trends in people’s choice of top-three concerned social issues. Social issues include health care, social security, education, corruption, employment, and income inequality. Data source: the Chinese Social Survey, 2006-20
